# Targeted transferosomal delivery of cetirizine: A new approach to alopecia management

**DOI:** 10.1371/journal.pone.0347648

**Published:** 2026-04-24

**Authors:** Noha Talal Zelai

**Affiliations:** Department of Biological Sciences, Faculty of Science, King Abdulaziz University, Jeddah, Saudi Arabia; National University of Rosario, ARGENTINA

## Abstract

**Introduction:**

Androgenetic alopecia is common in males and females; its prevalence increases with age. It affects the social life of individuals worldwide. The main problem associated with conventional minoxidil treatment is the risk of facial hypertrichosis. Cetirizine (CET), an antihistamine, may induce hair growth by increasing prostaglandin E2 expression. Therefore, Objectives: This study aimed to incorporate several CET formulations into transferosomes (TFS) and determine their optimal physicochemical properties. The optimal formulation was evaluated for its growth-promoting potential in vivo.

**Methods:**

The effect of CET-TFS was assessed by observing hair growth and quantifying insulin-like growth factor-1 (IGF-1) and vascular endothelial growth factor (VEGF) mRNA and protein levels in mice.

**Results:**

CET-TFS significantly enhanced hair growth in mice and stimulated high IGF-1 and VEGF mRNA and protein levels.

**Conclusion:**

These findings suggest that CET-TFS is a promising alternative to traditional hair growth medications. Additional toxicity and histopathological studies are required to confirm its suitability.

## Introduction

Androgenetic alopecia (AGA) is an age-related hair loss problem in men and women that has a substantial effect on the self-confidence and quality of life of affected individuals [1]. The prevalence of AGA increases from 30% to 80% in Caucasian men in their thirties and seventies, respectively [2,3]. A prevalence increase from 3% to 21% has been recorded in Caucasian women in their twenties and eighties, respectively [4,5]. Topical minoxidil is widely used to treat AGA. However, it is associated with several side effects, including facial hypertrichosis, headache, and pruritus [6]. Therefore, alternative treatments are required to overcome these problems.

Hair undergoes a three-phase cycle from the active and slow growth phases (anagen and catagen, respectively) to the regression phase, in which hair is shed for replacement [7]. Several prostaglandins affect the hair growth cycle. Prostaglandin E2 (PGE2) and prostaglandin F2A (PGF2A) promote hair follicle activity [8]. However, prostaglandin D2 (PGD2) levels increase before the regression phase. Additionally, topical application of PGD2 inhibits hair growth [9]. Hence, hair loss may be treated by promoting PGE2 and PGF2A production or suppressing PGD2.

Cetirizine (CET) was initially used for its pharmacological properties as an antihistamine. It blocks H1 receptors in the lungs and endothelial cells and stimulates the inflammatory response by activating mast cells and monocytes [10]. This effect results in PGE2 production and PGD2 inhibition [10,11]. Therefore, CET has substantial potential as an anti-AGA agent. Total hair density significantly increases after 16–26 weeks of 1% CET treatment, with no signs of sensitivity or irritation in treated patients [12,13].

Novel lipid-based nanodrug formulations of CET have improved its pharmacological properties. For example, elastic vesicle-based CET has shown improved results in dermatitis reduction and bioavailability prolongation compared with conventional formulations [14]. Similar improvements in efficacy and bioavailability with no observed side effects have been exhibited by lipid cubic systems [15], nanoemulsions [16], and niosomal nanocarriers [17]. These studies confirm the advantages of using CET in lipid nanocarriers to enhance topical delivery or treat dermatitis. However, to the best of our knowledge, no previous studies have examined the effects of CET lipid nanoformulations on AGA.

Transferosomes (TFS) are ultra-deformable lipid vesicles consisting of phospholipids and mild surfactants. These surfactants act as edge activators that help vesicles change their shape and squeeze through the spaces between skin cells while also maintaining structural integrity. This mechanism allows TFS to reach deeper skin layers, leading to higher penetration and drug accumulation abilities at the target sites compared with traditional liposomes [18,19]. Among the various elastic vesicular systems, including ethosomes, niosomes, proniosomes, and electrosomes, TFS have a notable ability to penetrate skin pores. Additionally, they offer several advantages, including the capacity to encapsulate hydrophilic and hydrophobic molecules, prolonged bioavailability, and reduced toxicity [18].

Recently, TFS have been used in several studies as nano-based carriers for anti-alopecia medications. The entrapment of minoxidil [20,21], dutasteride [22], finasteride [23], and *Phyllanthus emblica* plant extract [24] in TFS results in a highly controlled release and better therapeutic effect than the conventional formulation. TFS have not been investigated as carriers of CET for AGA treatment. Therefore, we aimed to prepare, characterise, and investigate the effects of a novel CET-TFS formulation on hair growth. This aim was successfully achieved, as indicated by the enhancement of mouse hair and upregulation of related indicators.

## Materials and methods

### Preparation method

Free CET was prepared by dissolving 40 mg of CET (cat No. A535405; AmBeed, Arlington, Texas, USA) in an equivalent volume of 10 mM phosphate-buffered saline (PBS; pH = 7.4). To prepare the TFS, Lecithin (Neogen Cochin, Kerala, India), Tween 80, and Span 80 (Loba Chemie, Mumbai, India) were dissolved in chloroform. Chloroform was subsequently evaporated above the lipid transition temperature under reduced pressure using a rotary evaporator (D-Lab with a water bath, Walnut, California, USA) to form a lipid film on the flask wall. Next, the film was hydrated at 45 °C with 100 mL of PBS (pH = 7.4) containing CET and rotated at 150 rpm [25,26]. Liposomes were allowed to swell at 25 °C for 2 h. The resulting vesicles were sonicated for 30 min using a probe sonicator to uniformly reduce their size. The prepared TFS were stored in an airtight closed container until further analysis. In total, 10 CET-TFS formulations were prepared. The compositions of these formulations and molar ratios of the formulation constituents (represented as proportional component ratios based on the quantities used) are listed in Table 1.

**Table 1:** Formulation code and variable used in preparation of TFS system.

Formula	Lecithin (mg)	Cholesterol (mg)	Span 80 (mg)	Tween 80 (mg)	CET (mg)
F1	190	0	20	0	40
F2	190	0	8	12	40
F3	190	0	10	10	40
F4	190	0	12	8	40
F5	190	0	0	20	40
F6	180	10	20	0	40
F7	180	10	8	12	40
F8	180	10	10	10	40
F9	180	10	12	8	40
F10	180	10	0	20	40

CET, cetirizine; TFS, transferosomes.

### Determination of loading efficiency (LE) and capacity (LC)

A calibration curve was prepared for CET, ranging from a concentration of 50–1 μg mL^-1^ in PBS (pH 7.4). A stock solution of CET in PBS (1:1; v/v) was prepared. Serial dilutions were prepared using methanol (Millipore; Merck, Billerica, co Wicklow, USA). Absorption maxima at 231 nm were measured at different drug dilutions.

The encapsulated CET was analysed using a UV-Vis spectrophotometer (Cary Series UV-Vis, NIR, Australia) and a standard curve. LE and LC were calculated using the following formulae [27]:


LE = [(initial concentration − free concentration) / initial concentration] × 100

LC = amount of total entrapped drug / total nanoparticle weight.

### Zeta potential and polydispersity index (PDI)

Dynamic light scattering (DLS: Zetasizer Nano ZN, Malvern Panalytical Ltd, United Kingdom) at a fixed angle of 173° at 25 °C was used to determine the zeta potential and PDI. The samples were sonicated, vortexed for 10 min, and diluted 10 times with deionised water before measurement [20]. Each sample was analysed in triplicate.

### Size and shape

Transmission electron microscopy (TEM; JEOL JEM-2100, USA) was performed at an accelerating voltage of 200 kV [20].

A droplet of the dissolved sample was placed on a Formvar carbon-coated 300-mesh copper grid (Ted Pella) and allowed to evaporate under ambient conditions. Size distribution and average size were determined using the Digital Micrograph 3 software (Gatan Inc., USA).

### In vitro release study

To study in vitro release, free CET and CET-TFS solutions and CET and CET-TFS gels in 1% w/v Carbopol 940 were prepared. Samples (5 mL) were loaded onto a dialysis membrane (Mwco 12–14 Kda) and suspended in 50 mL of PBS (pH = 7.4). At predetermined time points, 3 mL of the PBS was replaced with a fresh 3 mL of PBS. The samples were centrifuged for 5 min at 20,000 × g. The separated supernatant was analysed by spectrophotometry at 231 nm; the cumulative release percentage was calculated [28].

### In vivo study

Healthy male BALB/c mice (n = 40) weighing 25–35 g were randomly assigned to four groups. The negative control received 0.1 mL of intraperitoneally (IP) administered corn oil three times per week for two weeks. All other mouse groups received an IP dose of 0.1 mL testosterone three times per week for two weeks to induce alopecia [29]. Dorsal sides of all the mice were shaved. The positive control mouse model of alopecia was left untreated. Successful model induction was confirmed by visual observation of reduced hair regrowth in the testosterone-treated group (positive control) compared with that in the negative control. The last two groups were alopecia models that were topically treated with 1% CET and 1% CET-TFS gels daily for three weeks. Hair thickness of all the mice was qualitatively assessed using standardised digital photographs captured with a digital camera from the same dorsal skin area of each mouse on days 1, 5, 10, and 15 after treatment. The images were visually compared with those of the positive and negative control groups to evaluate relative differences in hair thickness. The assessment was performed under consistent lighting and positioning conditions to maintain comparability [20].

This study was conducted and reported in accordance with the ARRIVE guidelines for animal research and was approved by the University Research Animal Facility-Institutional Animal Care and Use Committee (approval number: URAF F 3 25).

### Quantitative reverse transcription polymerase chain reaction (qRT-PCR)

Total RNA was extracted using the TRIzol reagent (Cat# 15596026, Thermo Fisher Scientific). A LightCycler FastStart DNA Master SYBR Green I kit (Cat # 03003230001, Roche Life Science) was used for qRT-PCR to quantify the DNA. Extracted RNA samples (1 µg) were mixed with a buffer containing 10 mM Tris, 5 mM MgCl_2_, and 50 mM KCl (pH = 8.3). The mixtures were subsequently combined with an oligo dT-adaptor primer, RNase inhibitor, reverse transcriptase, and dNTPs. These mixtures were incubated for 15 min at 42 ºC and subsequently 5 min at 95 ºC to obtain 10 µL of cDNA.

DNA (2 µL) was mixed with the same amount of reaction mixture. Next, 10 pmol of each primer was added to obtain a final volume of 20 µL. Primers and PCR conditions are listed in Table 2 [30].

**Table 2:** Primers and quantitative reverse transcription polymerase chain reaction (qRT-PCR) conditions.

	Insulin-like growth factor-1 (IGF-1)	Vascular endothelial growth factor (VEGF)	Glyceraldehyde-3-phosphate dehydrogenase (GAPDH; control gene)
**Primer**	5′-CTGGTCCTGTGTCCCTTTGC-3 5′-GGACGGGGACTTCTGAGTCTT-3)	5′-CAACTTCTGGGCTCTTCTCG-3 5′-CCTCTCCTCTTCCTTCTCTTCC-3	5′-TGCACCACCAACTGCTTAGC-3, 5′-GGCATGGACTGTGGTCATGAG-3
**qRT-PCR conditions**			
**Denaturation**	95 ºC for 10 mins, 50 cycles (95 ºC for 10 s)		
**Annealing** **for 10 s**	66 ºC	64 ºC	66 or 64 ºC
**Extension**	72 ºC for 5 s		
**Calculation method**	mRNA expression was calculated by removing threshold cycles for the control gene and *IGF-1*	mRNA expression was calculated by removing threshold cycles for the control gene and *VEGF*.	

### Measurement of insulin-like growth factor-1 (IGF-1) and vascular endothelial growth factor (VEGF) protein levels

The mice were sacrificed using exsanguination under anaesthesia with a ketamine-xylazine cocktail 15 d after treatment. Hair follicles were collected from 2 × 2 cm treated areas of each group.

IGF-1 and VEGF protein levels in hair bulbs were measured using a Mouse & Rat IGF-I/IGF-1 ELISA Kit (Quantikine; R&D systems, Minneapolis, MN, USA) and a Mouse VEGF ELISA Kit (Quantikine; R&D systems), respectively, according to the manufacturer’s instructions [30]. Bulb samples were homogenised in purified water and centrifuged at 4 ºC for 20 mins at 20,400 × g to obtain supernatants. The supernatants were placed in monoclonal antibody–coated wells, incubated for 2 h at 25 ºC, and washed five times with 10 mM wash buffer (pH = 7.4). Next, conjugate reagents (IGF-1 or VEGF antibodies) were added; the plates were incubated for 2 h and washed five times. Detection reagents were added; the plates were incubated for 30 min at room temperature. The results were measured within 30 min using a microplate reader at a wavelength of 450 nm.

### Statistical analysis

All characterisation data related to zeta potential, DLS, and PDI are expressed as mean ± standard deviation. Significant differences in the drug release profiles and mRNA and protein expression data among groups were estimated using analysis of variance (ANOVA) and post-hoc Tukey’s tests. Statistical significance was set at *p* < 0.05. The sample size (n = 10 per group) was selected based on previous in vivo AGA studies [20,29]. This number was considered sufficient as it exceeded the sample size used in these studies (n = 5 [20] and n = 6 [29]) and was adequate to detect biologically relevant differences while also adhering to the ethical standards for animal experimentation.

## Results

### Characterisation of CET-TFS formulations

In total, 10 CET-TFS formulations were prepared. The zeta potential, zeta size, PDI, LE, and LC values are listed in Table 3.

**Table 3:** Characterisation of the prepared formulations.

Formula	Zeta potential (mV)	Average size (nm)	PDI	LE (%)	LC
F1	−39.2 ± 2.20	192.9 ± 21.69	0.409 ± 0.132	79.0612	15.81224
F2	−38.0 ± 1.80	167.9 ± 1.901	0.299 ± 0.032	54.72607	10.94521
F3	−27.6 ± 1.98	128.9 ± 3.137	0.248 ± 0.044	76.64355	15.32871
F4	−43.9 ± 0.850	189.4 ± 11.57	0.366 ± 0.084	63.71619	12.74324
F5	−30.2 ± 2.82	134.5 ± 5.314	0.519 ± 0.101	61.69998	12.34
F6	−43.6 ± 3.11	231.8 ± 9.246	0.591 ± 0.088	69.09908	13.81982
F7	−37.2 ± 2.19	156.3 ± 7.242	0.438 ± 0.108	67.95354	13.59071
F8	−34.5 ± 2.33	212.0 ± 12.86	0.287 ± 0.015	73.54976	14.70995
F9	−23.2 ± 2.23	181.3 ± 5.112	0.321 ± 0.047	61.19348	12.2387
F10	−29.4 ± 2.55	178.0 ± 0.000	0.30 ± 0.009	76.933507	15.38701

LC, loading capacity; LE, loading efficiency; PDI, polydispersity index.

F8 was the most suitable formulation, as shown in Table 3. It had a high stability, as evidenced by its high zeta potential (−34.5 ± 2.33), LE (73.54976), and LC (14.70995), an accessible size (212.0 ± 12.86), and monodisperse particles (0.287 ± 0.015).

**Fig 1:**
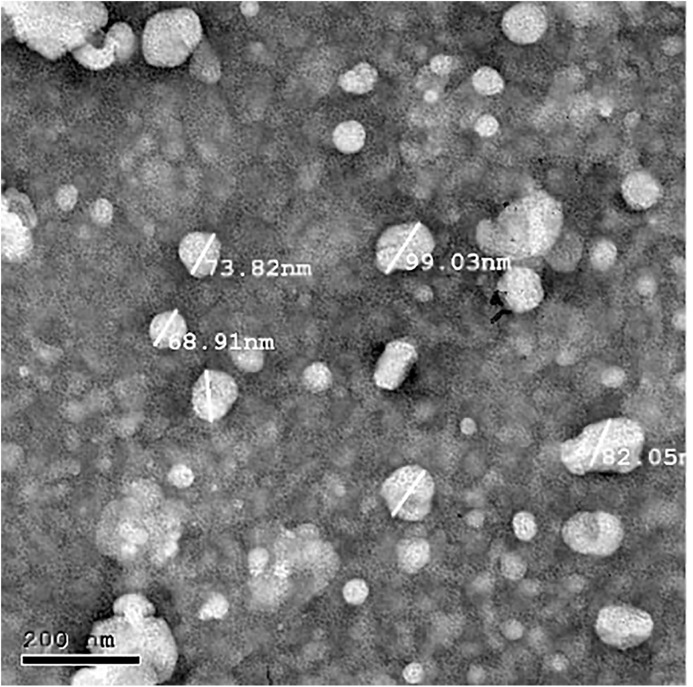
Transmission electron microscopy image of F8. nm; nanometre.

F4 and F6 possessed the highest zeta potential (*P* < 0.0377), although these were not significantly different from that of F8 (*P* = 0.0018 and *P* = 0.0026, respectively). Large particle sizes were observed in F6 and F8 compared with that in other formulations; however, only F6 exhibited significant differences (*P* ≤ 0.0024). Formulations with a low PDI (< 0.32) included F2, F3, F8, and F9; however, F3 only significantly differed from F6 (*P* = 0.0056). For the F8 formulation, TEM imaging showed an average particle size of 80.9 ± 11.4 nm (Fig 1).

### Release profile

**Fig 2:**
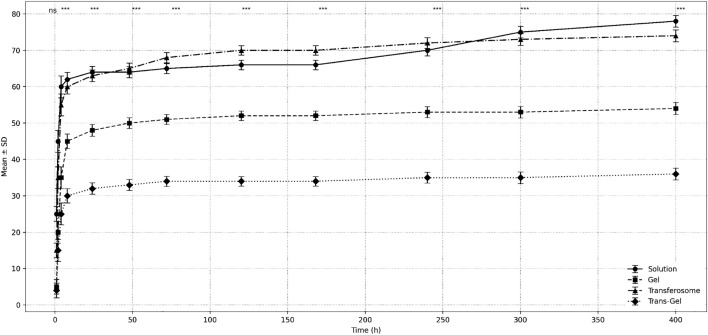
The cumulative release of F8 in phosphate-buffered saline. CET, cetirizine; TFS, Transferosomes.

The release profiles of all formulations followed a biphasic pattern, with an initial burst in the first 8 h followed by a steady increase, suggesting that CET release from the TFS gel was primarily governed by diffusion-controlled mechanisms. This behaviour is consistent with the Higuchi release model, which is commonly reported for vesicular and matrix-based delivery systems. ANOVA showed that at every time point, at least one formulation exhibited a slower release than the others (*P* < 0.00001). The release results (Fig 2) showed that the CET solution and CET-TFS exhibited the fastest release, with approximately 80% and 75% of the drug released by the 16^th^ day, respectively (*P* = 0.0609). These two formulations exhibited similar release patterns, as evidenced by non-significant differences in *P* values at the early time points of 4 h (*P* = 0.2503) and 8 h (*P* = 0.6298) and late time points of 240 h (*P* = 0.4141) and 300 h (*P* = 0.4642). The CET gel exhibited a nearly constant pattern, with 50–55% release from days 2–16. The slowest release was observed for the CET-TFS gel (*P* < 0.0001 compared with all other groups), with a constant release of 35% from days 2–16. ANOVA revealed that within the first 2 h, the CET solution exhibited a significantly higher release than the other groups (*P* < 0.0149).

Overall, the CET-TFS gel exhibited a favourable release rate. Therefore, they were used in subsequent studies. Zeta size analysis of CET-TFS revealed that it had a relatively large size of 496.6 ± 8.7 nm, high zeta potential of −65.0 ± 0.651, and PDI of 0.465 ± 0.056.

### Effect of the CET-TFS gel on hair growth

**Fig 3:**
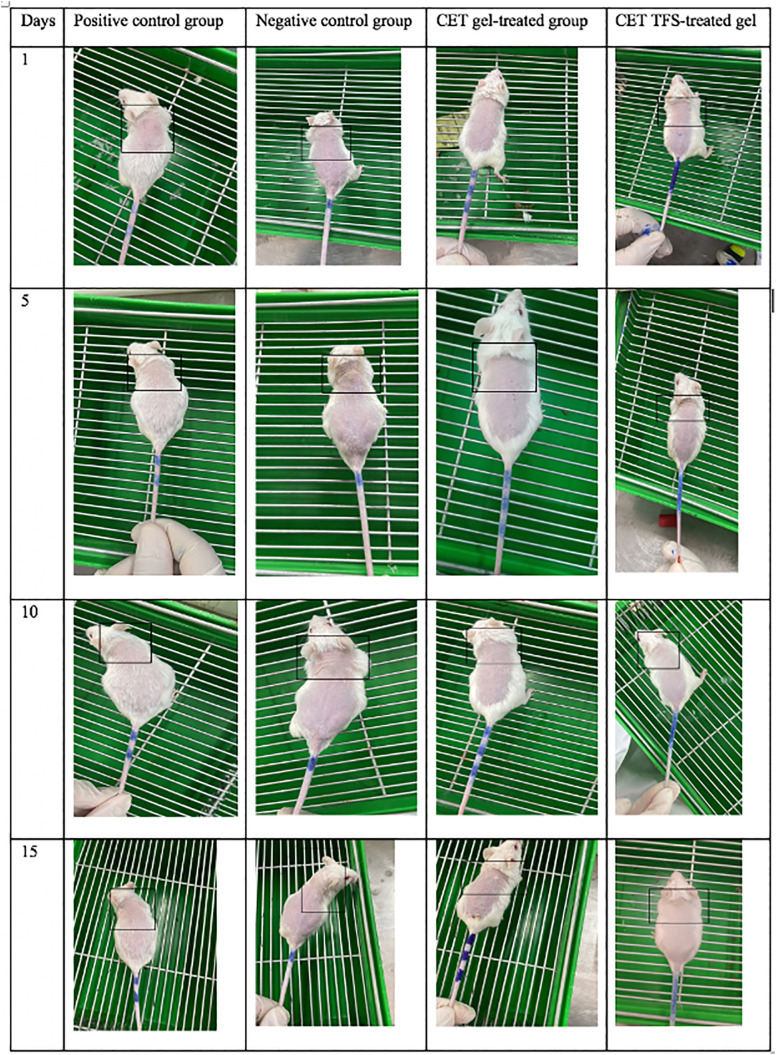
Hair thickness in different mice groups (n = 10 in each group). CET, cetirizine; TFS, Transferosomes.

**Fig 4:**
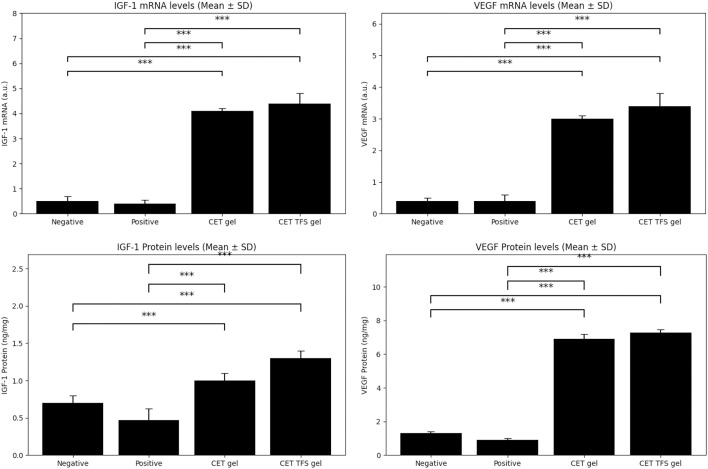
Insulin-like growth factor-1 (IGF-1) and vascular endothelial growth factor (VEGF) mRNA and protein expression levels in different groups.

The in vivo study (Fig 3) showed that topical application of CET gel resulted in hair thickness similar to that in the positive control group. CET-TFS gel–treated mice exhibited hair thickness similar to that in the CET gel and positive control groups during the first 5 d. However, by day 15, the CET-TFS gel group exhibited increased hair thickness compared with the negative control group (Fig 3), with elevated IGF-1/VEGF (Fig 4; *P* <<0.0001). Additionally, CET-TFS gel treatment resulted in elevated IGF-1 and VEGF levels; however, these levels were not significantly increased compared with those of the CET gel group (*P* ≥ 0.0515).

ANOVA showed that IGF-1 mRNA and protein expression levels in mice treated with CET and CET-TFS gel formulations were higher than those in the negative and positive AGA-untreated groups (*P* < 0.001). The two CET-treated groups exhibited no statistically significant differences (*P* = 0.338 and *P* = 0.0515 for mRNA and protein, respectively).

Similarly, mRNA and protein VEGF expression levels were higher in the CET formulations than in the negative and positive control groups (*P* < 0.0001). Although these levels were higher in the CET-TFS-treated group than in the CET-treated group, the increases were not significant (*P* = 0.1897 and *P* = 0.179 for mRNA and protein, respectively).

## Discussion

In this study, different CET-TFS formulations were prepared, among which the best formulation was tested in water and gel vehicles for favourable release; the chosen formulation was tested to increase mouse hair thickness.

Zeta potential is a critical indicator of the stability of nanoparticle formulations. Values greater than 30 mV indicate low aggregation and high electrostatic repulsion between particles [31]. Therefore, formulation stability increases with zeta potential. Conversely, low PDI values indicate a narrow size distribution. Hence, favourable formulations were selected based on their stability and homogeneity, which were expressed as the highest zeta potential and lowest PDI [32].

Among the prepared formulations, F4 and F6 exhibited higher zeta potential (−43.9 ± 0.850 and −43.6 ± 3.11 mV, respectively) than F8 (−34.5 ± 2.33). However, these formulations possessed higher PDI (0.366 ± 0.084 and 0.591 ± 0.088, respectively) than F8 (0.287 ± 0.015). Previous studies have reported that a PDI < 0.3 indicates uniform particles [32]. F4 and F6 possessed lower LE (63.7 and 69.1, respectively) and LC (12.7 and 13.8, respectively) values than F8 (LE: 73.5; LC: 14.7). F8 was chosen because of its superior physicochemical properties.

The average zeta size for F8 using DLS (212.0 ± 12.86) was higher than that of the average size observed using TEM 80.9 ± 11.4 nm. TEM lacks the hydrodynamic diameter [33].

The selected formulation, F8, was established as most suitable vehicle for the CET-TFS solution and gel. F8 exhibited a high zeta potential of −34.5 ± 2.33 mV, which indicates a high stability. Notably, the CET-TFS gel exhibited an even higher zeta potential of −65.0 ± 0.651 mV, suggesting superior stability of the gel formulation.

In previous studies, entrapment of CET resulted in a substantially smaller particle size of 32 nm in nanoemulsion gels [16], 139.7 nm in elastic vesicle-based systems [14], and 147.1 to 153 nm in lipid cubic systems [15] compared with 496.6 nm of the TFS gel in this study, which had a size comparable to that of a previous niosomal nanocarrier (403.4 nm) [17]. Our CET-TFS gel possesses superior stability, as evidenced by a high zeta potential (−65.0) compared with 12.73 in elastic vesicle-based systems [14], −12.9 ± 1.7 in niosomal nanocarriers [17], –15.5 ± 0.2–2.1 in lipid cubic systems [15], and –19.31 in nanoemulsion gels [16].

Although DLS measurements showed that the CET-TFS gel had a relatively larger size of 496.6 ± 8.7 nm and higher PDI values (0.465 ± 0.056) and zeta potential (−65.0 ± 0.651), it had a slower release profile than other formulations. The in vitro release study showed that the CET gel had a sustained release pattern, consistently between 50% and 55% release from 2 d to 2 weeks. This slow release can be attributed to the polymer network, which creates diffusion barriers. Unlike drug solutions, drug molecules are forced to travel through a three-dimensional matrix comprising a viscous medium. Hence, drug molecules exhibit a slow diffusion rate and reduced molecular mobility [34].

The CET-TFS gel demonstrated the slowest release, with a sustained release of 35% from day 2–12. This slow release can be attributed to the integration of drug diffusion through the TFS and gel network, which causes non-Fickian release [35]. Such sustained release profiles are considered advantageous as they maintain therapeutic drug levels for an extended time and reduce dosing frequency, thereby guaranteeing patient commitment to the drug regimen [36]. This behaviour may be attributed to presence of the gel matrix, which prevents drug diffusion [37]. However, the CET solution and CET-TFS released approximately 80% and 75% of the drug within 14 d, respectively. Previous studies have shown an accelerated release profile of 50–75% after 24 or 48 h in lipid cubic systems [15], 98.5% after 1 h in nanoemulsion gels [16], and 80% after 12 h in niosomal nanocarriers [17].

After selecting best properties of the CET formulation, its effect on AGA was assessed. In vivo studies indicated that mice treated with CET-TFS gel exhibited hair thickness comparable to that of testosterone-injected mice and CET-treated mice on days 1 and 5. However, on days 10 and 15, hair thickness in the CET-TFS gel-treated group surpassed that of the negative control group, which was not treated with testosterone. These results were consistent with the observed IGF-1 and VEGF mRNA and protein levels, which were higher in CET-TFS gel–treated cells than in untreated cells, although the result was not significant. These two indicators are strongly associated with the activity of hair follicles as IGF-1 stimulates cell division and VEGF is involved in blood and nutrient supply around the follicles. A previous study has found that an increase in the expression of these two indicators is associated with high growth stimulation by minoxidil [30].

These findings suggest that the CET-TFS gel formulation has a promising effect in promoting hair growth, which may be attributed to enhanced drug stability and sustained release. This study has some limitations associated with the lack of positive comparators, such as minoxidil, and the use of only male murine models, which may not reflect the effects in females or humans.

## Conclusions

Among the other tested formulations, F8 of the CET-TFS displayed favourable physicochemical characteristics, including high stability, low dispersity with high LE and LC, and a suitable particle size. Constant drug release of the CET-TFS gel and high in vivo hair thickness further indicate that it may represent a promising approach for topical CET delivery in AGA management. These findings are consistent with those of previous studies on nanoparticle-based drug delivery systems. However, this study has some limitations, including the lack of drug toxicity and histopathological evaluations, a validation of the testosterone-induced alopecia model, a positive treatment comparator, such as 2% and 5% minoxidil, and a quantitative measurement of hair thickness.

## Supporting information

S1 File CET data. (CSV)
